# Supplementary figures and images for: TPL2 kinase activity regulates microglial inflammatory responses and promotes neurodegeneration in tauopathy mice
Source: eLife. 2023 Aug 9;12:e83451. doi: 10.7554/eLife.83451 (PMC10411973; doi:10.7554/eLife.83451)

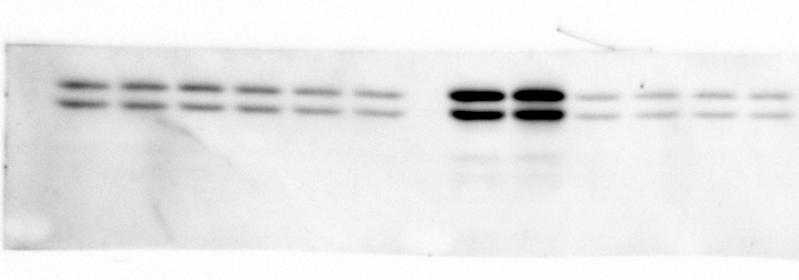

Supplement: Figure 1—source data 1. [file elife-83451-fig1-data1.zip › Figure 1-source data/p-ERK image.tif]

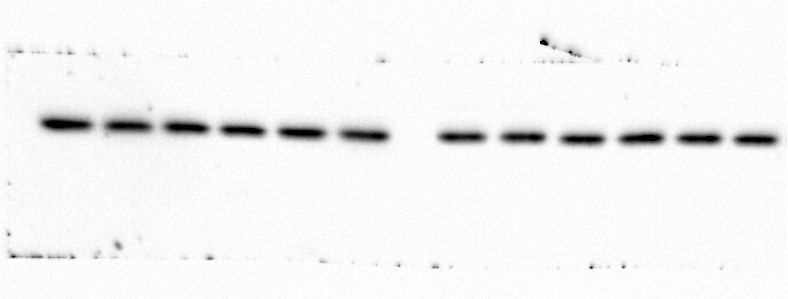

Supplement: Figure 1—source data 1. [file elife-83451-fig1-data1.zip › Figure 1-source data/p38 image.tif]

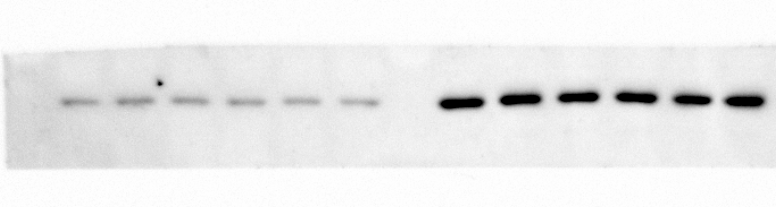

Supplement: Figure 1—source data 1. [file elife-83451-fig1-data1.zip › Figure 1-source data/p-p65 image.tif]

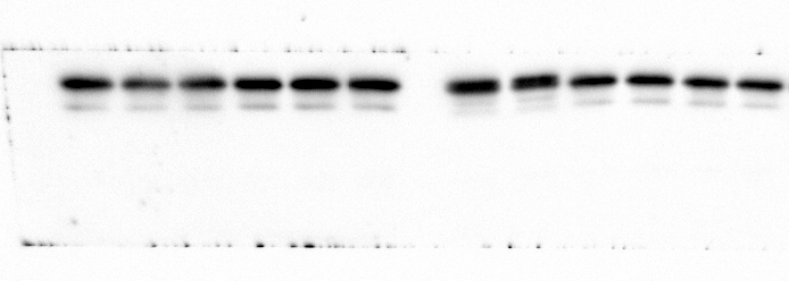

Supplement: Figure 1—source data 1. [file elife-83451-fig1-data1.zip › Figure 1-source data/ERK_image.tif]

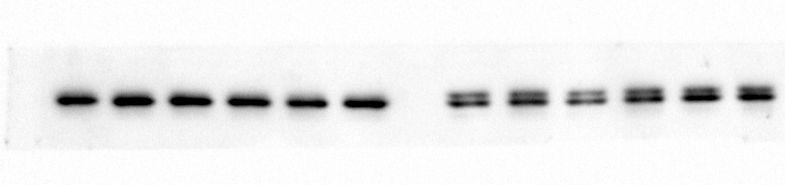

Supplement: Figure 1—source data 1. [file elife-83451-fig1-data1.zip › Figure 1-source data/p65 image.tif]

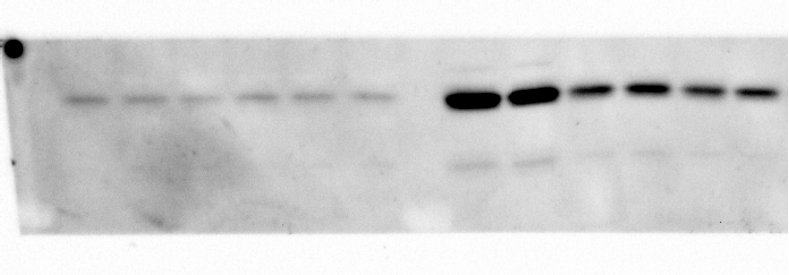

Supplement: Figure 1—source data 1. [file elife-83451-fig1-data1.zip › Figure 1-source data/p-p38 image.tif]

Figure 1A source data

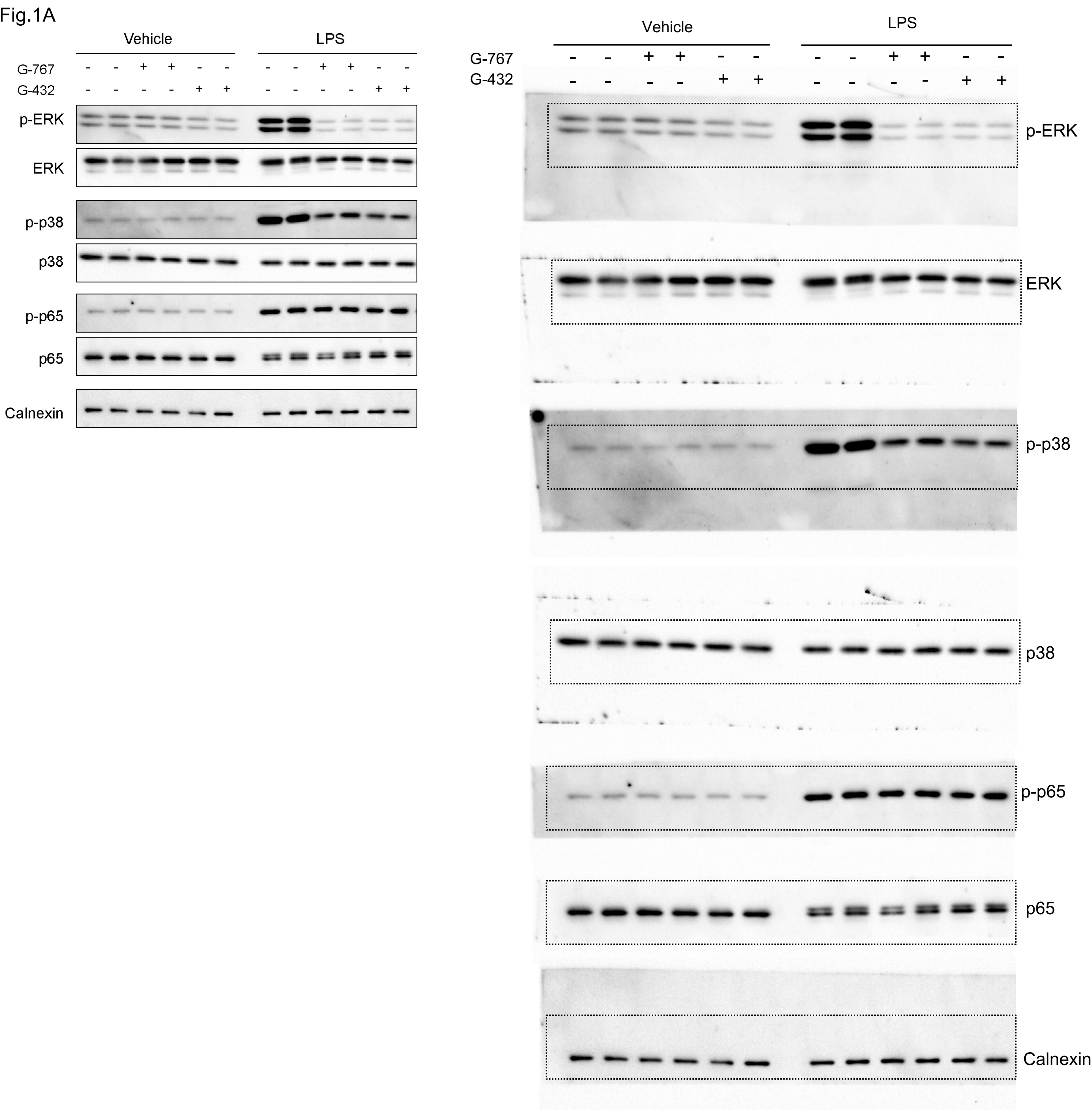

Fig. 1A - source data.  
Full images of the western blots.

Supplement: Figure 1—source data 1. [file elife-83451-fig1-data1.zip › Figure 1-source data/Figure 1A source data.pdf]

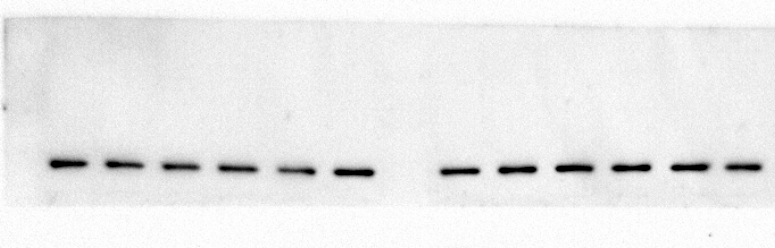

Supplement: Figure 1—source data 1. [file elife-83451-fig1-data1.zip › Figure 1-source data/calnexin image.tif]

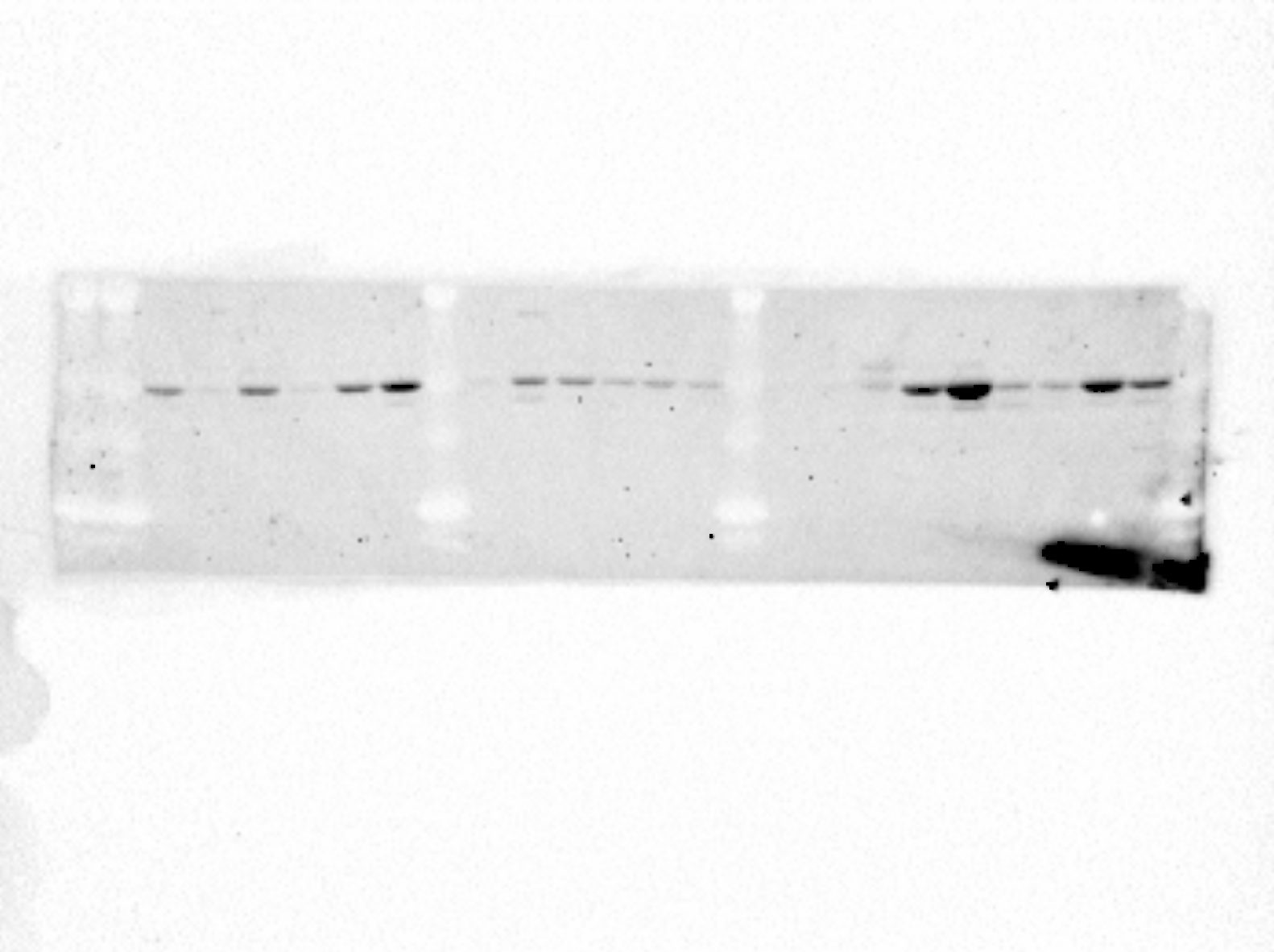

Supplement: Figure 1—figure supplement 1—source data 1. [file elife-83451-fig1-figsupp1-data1.zip › Figure1-figure supplement 1-source data/TPL2 raw image.tif]

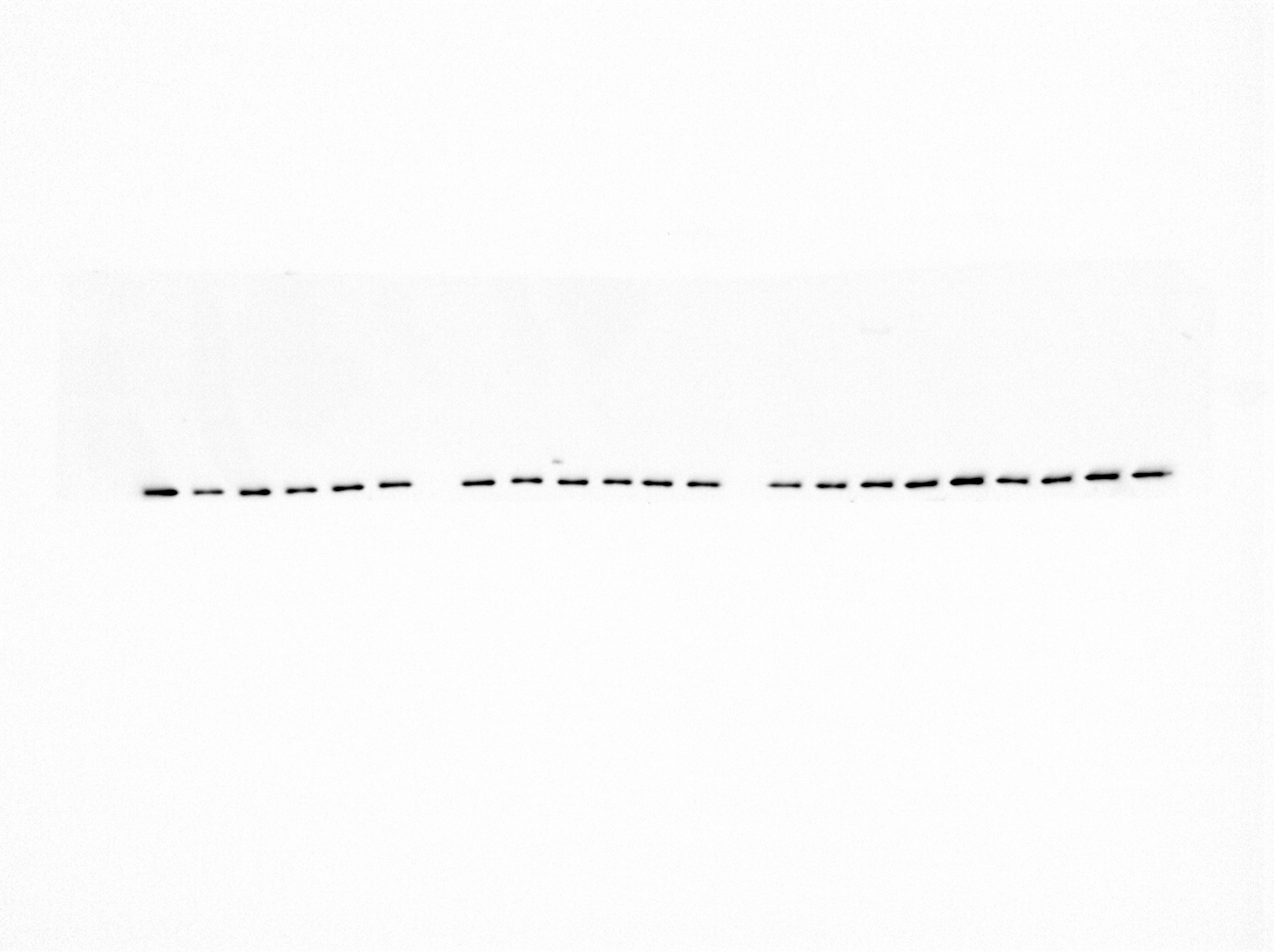

Supplement: Figure 1—figure supplement 1—source data 1. [file elife-83451-fig1-figsupp1-data1.zip › Figure1-figure supplement 1-source data/Calnexin raw image.tif]

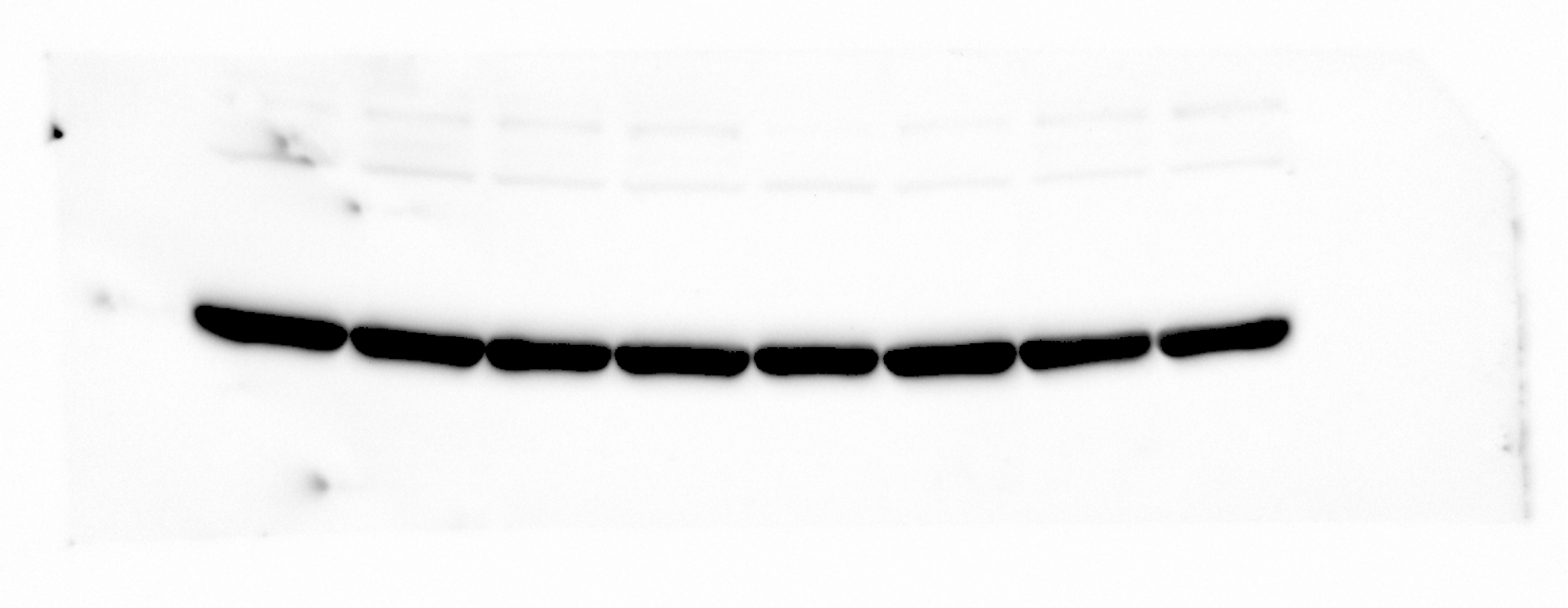

Supplement: Figure 2—source data 1. [file elife-83451-fig2-data1.zip › Figure 2-source data/Fig2C_Actin image.tif]

Figure 2C and 2D source data

Fig. 2C

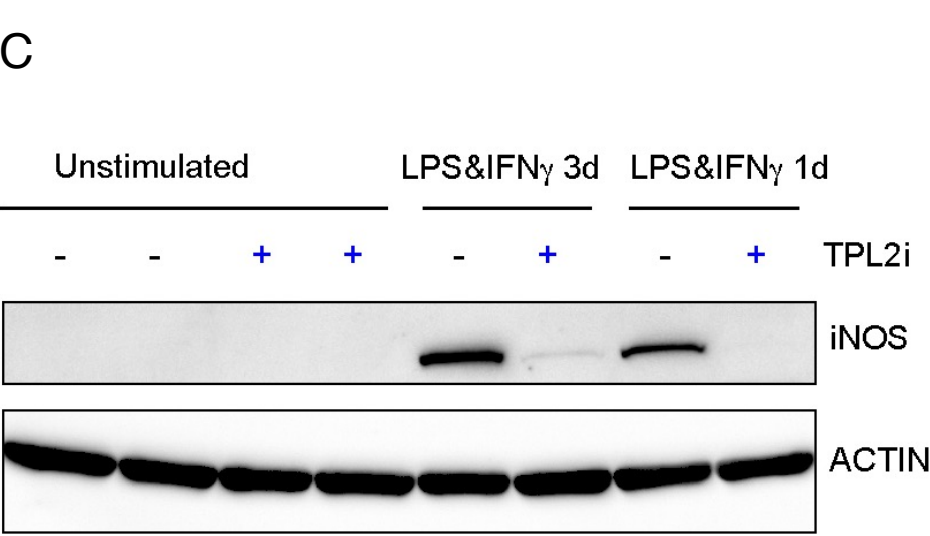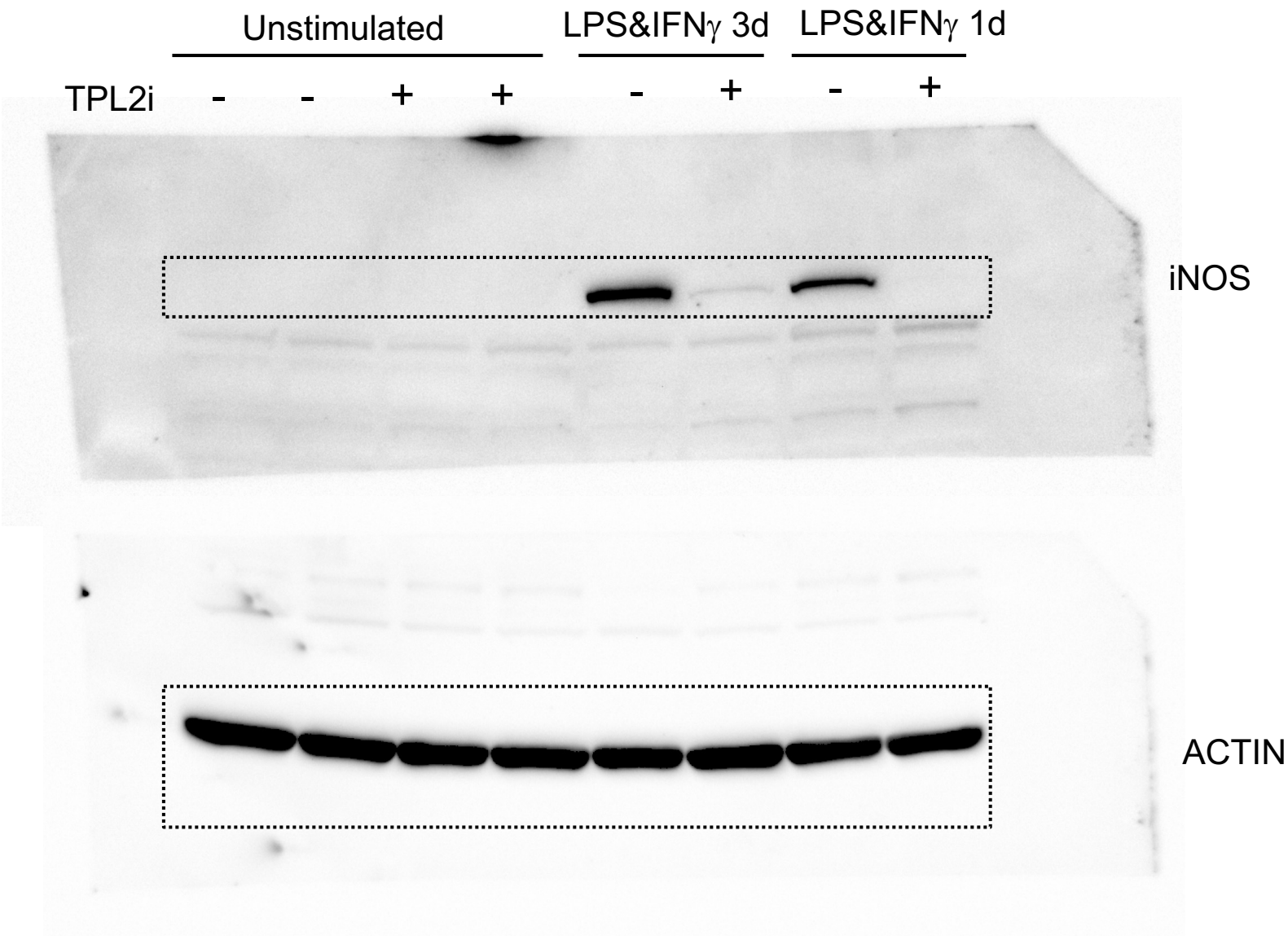

Fig. 2D

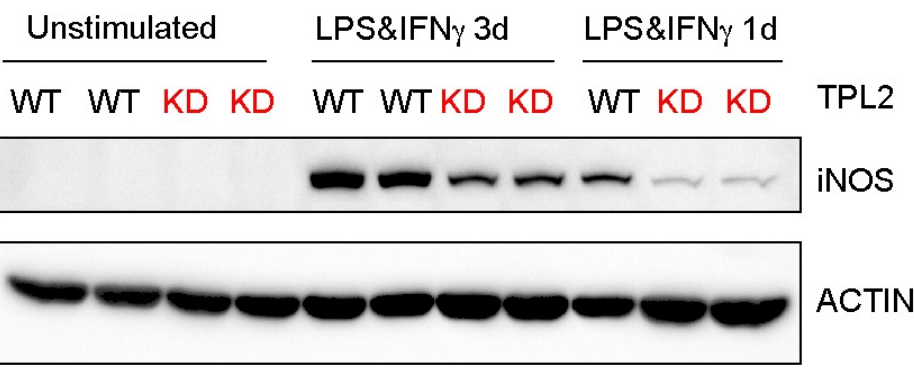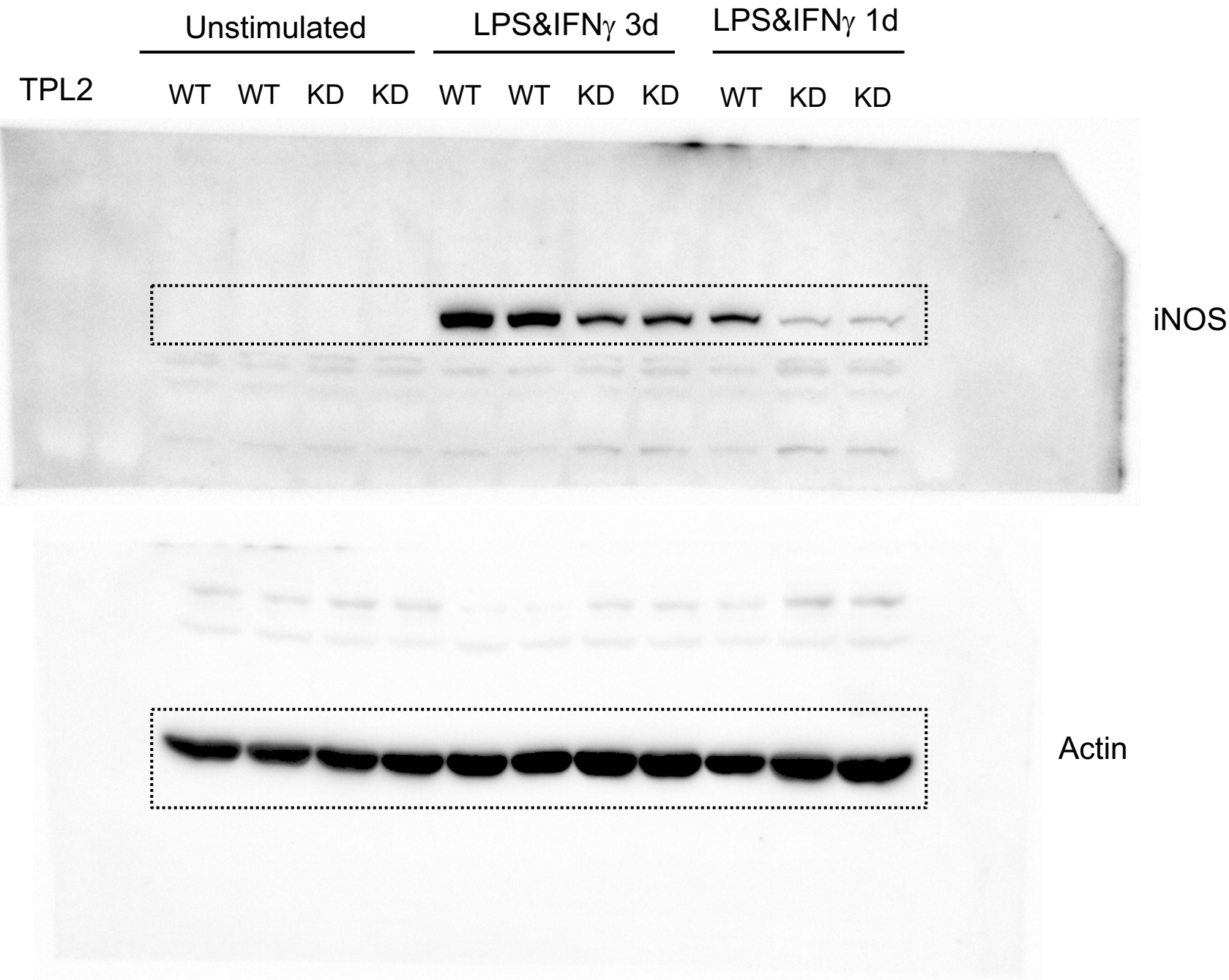

**Fig. 2 - source data.**  
Full images of the western blots.

Supplement: Figure 2—source data 1. [file elife-83451-fig2-data1.zip › Figure 2-source data/Figure 2C&D source data.pdf]

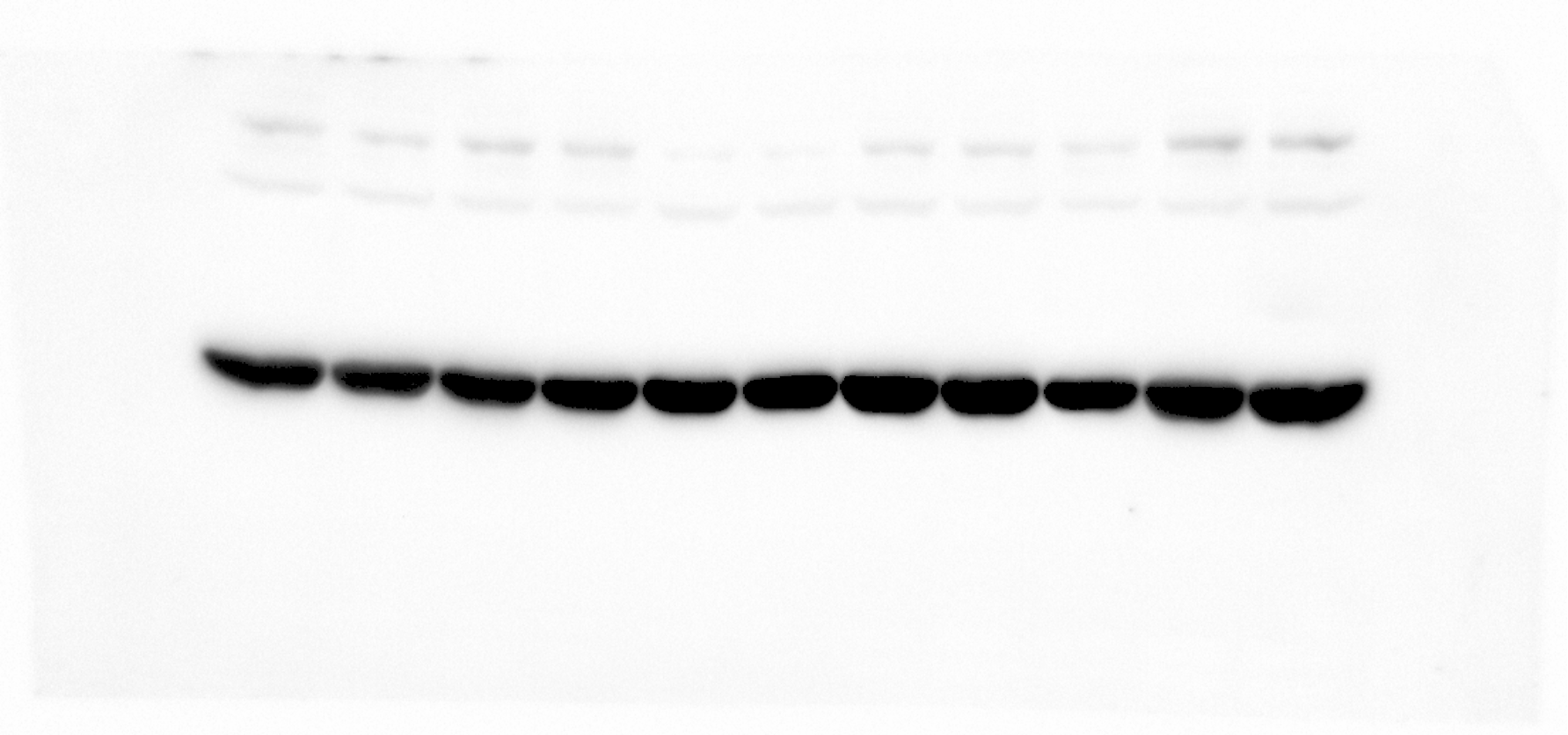

Supplement: Figure 2—source data 1. [file elife-83451-fig2-data1.zip › Figure 2-source data/Fig2D_Actin image.tif]

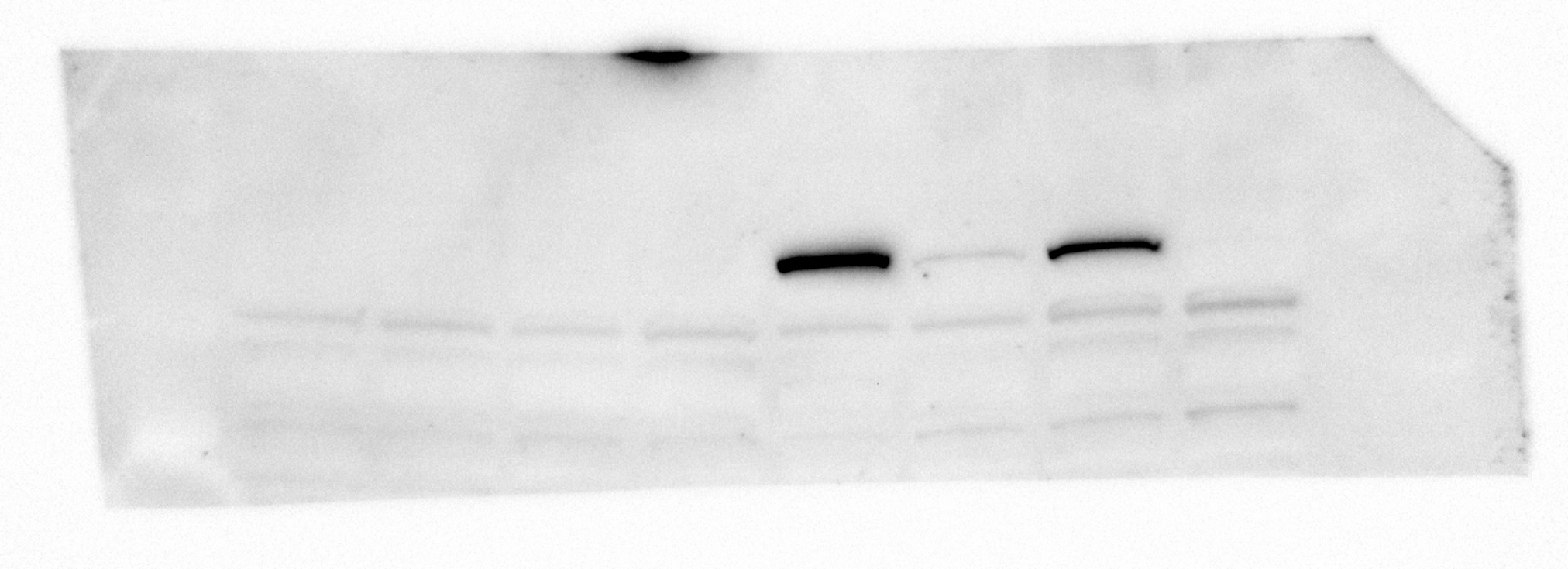

Supplement: Figure 2—source data 1. [file elife-83451-fig2-data1.zip › Figure 2-source data/Fig2C_iNOS image.tif]

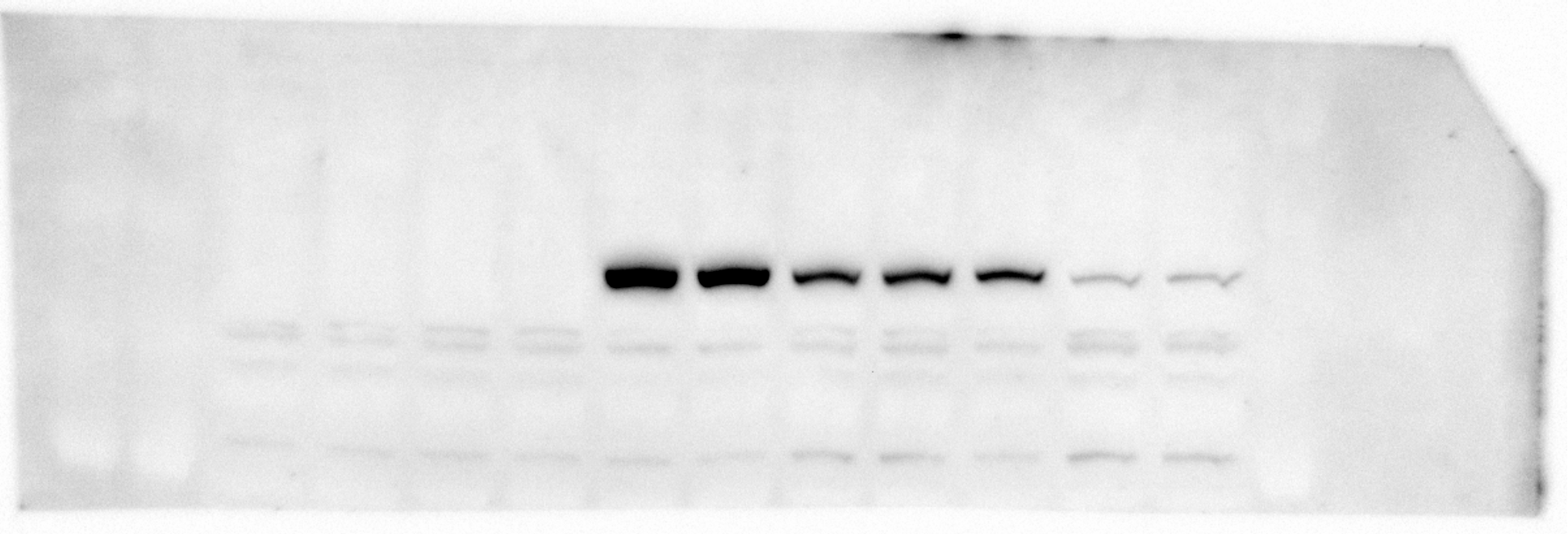

Supplement: Figure 2—source data 1. [file elife-83451-fig2-data1.zip › Figure 2-source data/Fig2D_iNOS image.tif]
