# Supplementary material for: TPL2 kinase activity regulates microglial inflammatory responses and promotes neurodegeneration in tauopathy mice
Source: eLife. 2023 Aug 9;12:e83451. doi: 10.7554/eLife.83451 (PMC10411973; doi:10.7554/eLife.83451)
Supplement: Figure 1—figure supplement 1—source data 1. [file elife-83451-fig1-figsupp1-data1.zip › Figure1-figure supplement 1-source data/Figure 1-figure supplement 1C source data.pdf]

Figure 1-figure supplement 1 source data

C

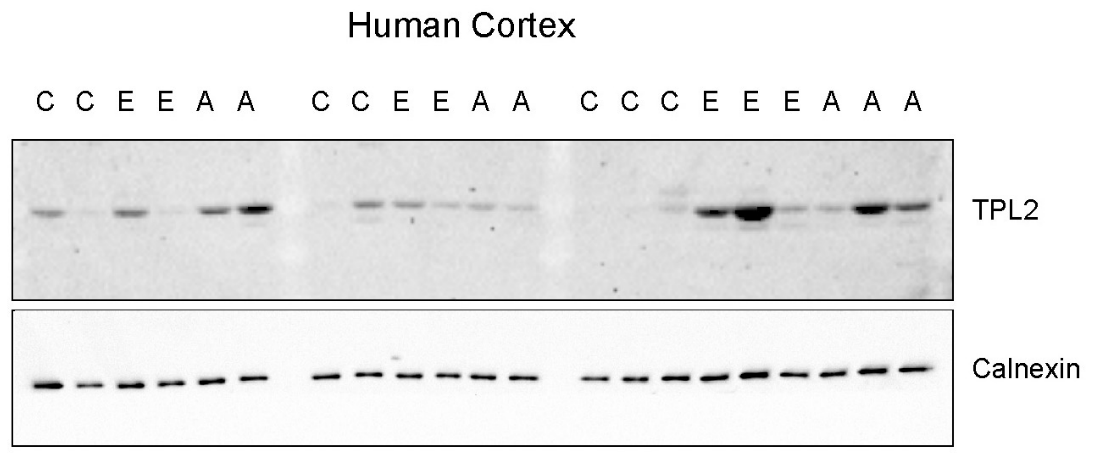

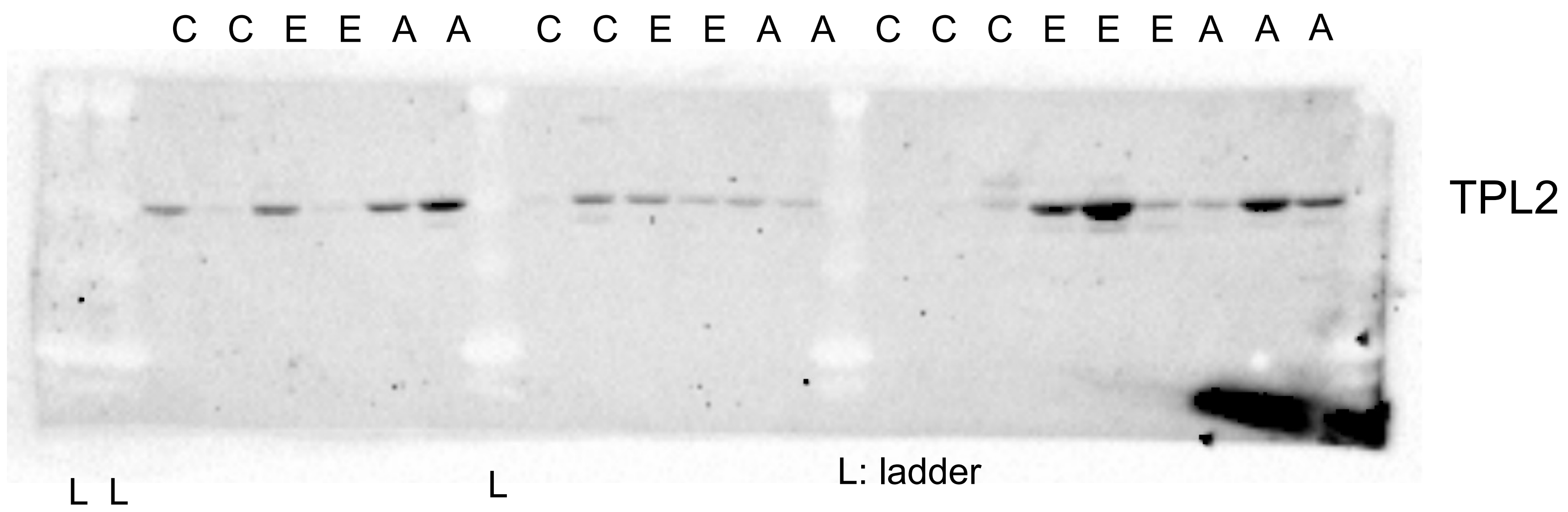

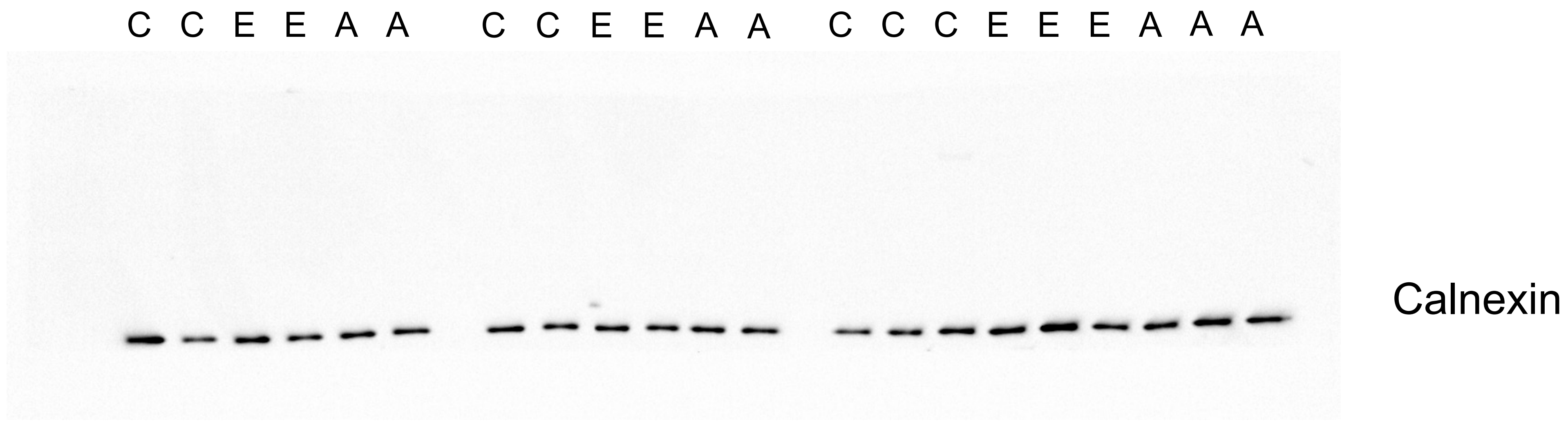

Figure 1-figure supplement 1C-source data.

Full images of the western blots.
